# Neuroimaging-Based Scalp Acupuncture Locations for Dementia

**DOI:** 10.3390/jcm9082477

**Published:** 2020-08-01

**Authors:** Jin Cao, Yiting Huang, Nathaniel Meshberg, Sierra A. Hodges, Jian Kong

**Affiliations:** Department of Psychiatry, Massachusetts General Hospital, Harvard Medical School, Building 120, 2nd AVE, Charlestown, MA 02129, USA; jcao9@mgh.harvard.edu (J.C.); yiting.h@hotmail.com (Y.H.); nmeshberg@mgh.harvard.edu (N.M.); sahodges@mgh.harvard.edu (S.A.H.)

**Keywords:** scalp acupuncture, neuroimaging, meta-analysis, fMRI, dementia, neuromodulation

## Abstract

Scalp acupuncture is a modality of acupuncture in which acupuncture needles are inserted into a certain layer of the scalp in order to affect the function of corresponding areas of the cerebral cortex and relieve symptoms. Clinical studies have demonstrated the potential of scalp acupuncture as a non-pharmacological treatment for dementia. Unfortunately, recent findings from brain neuroimaging studies on dementia have not been incorporated into scalp acupuncture. This study aims to integrate meta-analysis, resting-state functional connectivity, and diffusion tensor imaging (DTI) to identify potential locations of scalp acupuncture for treatment of dementia. We found that the prefrontal cortex, the medial prefrontal cortex, the middle and superior temporal gyrus, the temporal pole, the supplementary motor area, the inferior occipital gyrus, and the precuneus are involved in the pathophysiology of dementia and, therefore, may be the target areas of scalp acupuncture for dementia treatment. The neuroimaging-based scalp acupuncture protocol developed in this study may help to refine the locations for the treatment of dementia. Integrating multidisciplinary methods to identify key surface cortical areas associated with a certain disorder may shed light on the development of scalp acupuncture and other neuromodulation methods such as transcranial electrical current stimulation, particularly in the domain of identifying stimulation locations.

## 1. Introduction

In recent years, acupuncture has dramatically increased in popularity and has been widely used in clinical practice in both Eastern and Western societies due to its curative effects and small side effects. Many experimental and clinical studies have been performed to test acupuncture’s efficacy and/or investigate its underlying mechanisms. However, traditional acupuncture is based on the Meridian theory, which believes that meridians are the channels in which the blood and qi flow, and acupuncture produces a therapeutic effect by modulating the flow of qi and blood. The current meridian theory (particularly the distribution of the meridians in the human body) is based on the Yellow Emperor’s Classic of Internal Medicine, a classical text believed to have been written thousands of years ago, and since then, there has been no significant change in this meridian theory.

In recent decades, innovative acupuncture modalities, such as scalp acupuncture, auricular acupuncture, abdominal acupuncture, and wrist acupuncture, have been developed based on new theories. The clinical application and efficacy of these emerging acupuncture modalities have been widely applied in acupuncture practice and have become key components of modern acupuncture. One such modality, which has been attracting more and more attention, is scalp acupuncture.

Scalp acupuncture is a modality of acupuncture, where acupuncture needles are inserted into a certain layer of the scalp and believed to be able to modulate the brain neurons of the underlying areas. The unique characteristic of scalp acupuncture is that the stimulation locations of the needles are based on modern anatomy and neurophysiology, providing a foundation for its development.

Since the emergence of scalp acupuncture in the 1970s, the scope of application of scalp acupuncture has been constantly expanding, involving various disorders. For example, scalp acupuncture has been widely used in the rehabilitation of stroke patients, as it can directly stimulate the scalp areas corresponding to the motor, sensory and language cortices, thereby improving the symptoms of stroke patients accordingly [1,2,3].

In recent decades, brain imaging technologies such as magnetic resonance imaging (MRI) and positron emission tomography (PET) have been widely used to investigate the pathophysiology of brain disorders such as dementia, depression, insomnia, autism, and schizophrenia, and have significantly enhanced our understanding of the underlying mechanisms of these disorders [4]. Nevertheless, findings from these studies have not been incorporated into scalp acupuncture protocol. Incorporating the findings from cutting-edge brain imaging tools into scalp acupuncture may present a crucial next step for the development of scalp acupuncture.

Dementia, characterized by a deterioration in cognitive function beyond what might be expected from normal ageing, is one of the most common diagnoses that impact healthspan as people age. Dementia is a challenge to patients, caregivers, and healthcare providers, and carries a heavy financial burden, with the cost of caring for dementia patients estimated to rise to 2 trillion US dollars annually by 2030 [5]. Clinical studies have shown that acupuncture, especially scalp acupuncture, can significantly improve the clinical symptoms of patients with dementia [6,7,8]. However, no research has confirmed that the stimulation targets of scalp acupuncture for treating dementia are consistent with the neuroimaging findings of dementia.

Thus, this study will initiate an attempt to develop a neuroimaging-based scalp acupuncture prescription/protocol for treatment of dementia. Specifically, we will first apply a meta-analysis on brain imaging studies of dementia and summarize surface brain regions associated with dementia that can be modulated by scalp acupuncture. Then, we will apply resting-state functional connectivity and diffusion tensor imaging (DTI) to further identify potential surface regions that are functionally/anatomically connected to deep brain structures that play an important role in dementia (i.e., hippocampus) but cannot be directly modulated by scalp acupuncture. Finally, we will develop a scalp acupuncture prescription for dementia based on the findings achieved.

## 2. Methods

In this study, we applied several methods to identify potential locations for scalp acupuncture treatment of dementia. First, we used the meta-analysis on brain imaging studies of dementia (Method 1) to identify brain areas involved in dementia, particularly the surface brain regions associated with dementia. We then performed resting-state functional connectivity analysis (Method 2) to identify surface regions that are functionally linked to the hippocampus (a key deep brain structures associated with dementia) based on a cohort of subjects collected in our lab. We next applied DTI analysis (Method 3) on the same cohort of subjects as Method 2 to identify surface cortical regions that are anatomically connected with the hippocampus. Finally, we proposed a neuroimaging-based scalp acupuncture prescription based on the findings from these three methods.

### 2.1. Method 1: Identifying Dementia-Associated Surface Cortical Regions for Scalp Acupuncture Using Meta-Analysis

To extract dementia-associated brain regions, we used Neurosynth [9] (http://neurosynth.org/: accessed 16 April 2020) as a metadata reference for neuroimaging literature. Under the search string “dementia”, 142 fMRI studies were identified, and a uniformity test map was generated to identify dementia-associated brain regions. A complete list of the 142 fMRI studies extracted from Neurosynth can be found in Table S1.

Similar to our previous study [10,11,12], we first created a brain surface cortical mask using SPM Wake Forest University (WFU) PickAtlas toolbox (http://fmri.wfubmc.edu/software/pickatlas, version 3.0.5) to identify dementia-associated surface brain regions. The regions included in the brain surface cortical mask were the bilateral pre- and postcentral gyrus; superior and middle frontal gyrus; superior, inferior, and middle occipital gyrus; superior and inferior parietal lobules; supramarginal gyrus; angular gyrus; superior temporal gyrus; superior temporal pole; middle temporal gyrus; middle temporal pole; inferior temporal gyrus; opercular inferior frontal gyrus; Rolandic operculum; triangular inferior frontal gyrus; superior medial frontal gyrus; calcarine sulcus; orbital middle, superior, and inferior frontal gyrus; orbital medial frontal gyrus; supplementary motor area; paracentral lobule; precuneus; and cuneus [10,11,12]. Next, brain regions from the meta-analysis were further refined by taking the overlap of the uniformity test map with the brain surface cortical masks and then using the xjView toolbox (http://www.alivelearn.net/xjview) to identify the coordinates with peak z-scores within the all surface cluster larger than 30 voxels on the uniformity test map. Finally, we visually checked the locations of the brain regions obtained to identify potential brain surface regions that are accessible by scalp acupuncture.

### 2.2. Method 2: Identifying Dementia-Associated Surface Regions from the Resting-State Functional Connectivity Analysis

Although Method 1 identified some surface cortical regions that may be used for scalp acupuncture directly, some brain regions identified from the meta-analysis, such as the hippocampus, are located in “deep” areas inaccessible to scalp acupuncture.

Nevertheless, these regions may be functionally or anatomically connected to surface areas. Thus, we may influence the function of these deep brain structures indirectly by stimulating with scalp acupuncture surface areas that are connected with these deep structures [10,11,13]. Thus, in this study, we performed resting-state fMRI analysis (Method 2) and DTI analysis (Method 3) to further identify surface brain regions that are functionally or anatomically connected with key regions involved in dementia.

#### 2.2.1. Subjects and MRI Data Acquisition

MRI data from twenty-four healthy, right-handed individuals were used in this study. The Partners Institutional Review Board (IRB) of Massachusetts General Hospital approved the study. All subjects provided written informed consent prior to participating in the study.

All fMRI data were acquired at the Massachusetts General Hospital Martinos Center for Biomedical Imaging with a 3-axis gradient head coil in a 3 Tesla Siemens MRI System. A high-resolution T1-weighted structural image was acquired by an isotropic multiecho magnetization-prepared rapid acquisition with gradient echo (MPRAGE) pulse sequence for anatomic localization of significant signal changes. To obtain reliable resting state fMRI data, each subject participated in four scan sessions, each session separated by at least 7 days [14]. An 8 min resting-state fMRI scan was performed in each scan session, and subjects were asked to keep their eyes open and to blink normally while looking at a darkened screen and to not think about any particular thing during the scan. Functional images were acquired using a gradient echo T2*-weighted pulse sequence (time repetition (TR)/time echo (TE) = 2000/30 ms, flip angle (FA) = 90°, field of view (FOV) = 192 × 192 mm, 48 anterior and posterior commissure (AC-PC) aligned slices, slice thickness = 3.0 mm with 0.6 mm interslice gap, 90 image volumes per slice, and matrix size = 96 × 96) and a 32-channel multiarray coil.

#### 2.2.2. fMRI Data Preprocessing

Functional connectivity analysis was performed using the CONN toolbox version 18.b (http://www.nitrc.org/projects/conn). We used the default preprocessing pipeline for seed-to-voxel resting-state functional connectivity (rsFC) analysis. The specific steps were as follows: slice timing correction, head motion correction, skull-stripping using BET, co-registration of the anatomical image to the mean functional image, segmentation of the anatomical gray matter, white matter, and CSF, normalization to the Montreal Neurological Institute (MNI) standard coordinate space, and smoothing with a 6 mm Gaussian kernel. Band-pass filtering was performed with a frequency window of 0.008–0.09 Hz.

We selected the hippocampus as the region of interest (ROI) to conduct seed-to-voxel functional connectivity analyses (left and right hippocampus separately). We chose this region as it was also detected in the meta-analysis (Method 1). Additionally, the important role of the hippocampus in dementia has been well documented [15,16]. The ROIs were created using the WFU PickAtlas toolbox.

In the first-level analysis, we produced a correlation map for each subject by extracting the BOLD time course separately from the ROI and computing Pearson correlation coefficients between the time course in the ROI and every voxel of the whole brain. Correlation coefficients were Fisher transformed into ‘z’ scores to increase normality. In the group-level seed-to-voxel connectivity analysis, all subject-level seed maps were included in a one-sample *t*-test to obtain a group-level correlation map (positive and negative correlation separately). We also applied the brain surface cortical mask (the same surface mask as we used in Method 1) to exclude regions not located on the brain surface (inaccessible to scalp acupuncture stimulation). A threshold of voxel-wise *p* < 0.001 (uncorrected) and cluster-level *p* < 0.05 false discovery rate (FDR) corrected were applied.

### 2.3. Method 3: Identifying Dementia-Associated Surface Regions from the DTI Analysis

#### 2.3.1. Diffusion MRI Data Acquisition

Diffusion MRI (dMRI) data from twenty-four healthy, right-handed individuals were used in this study (same subjects as resting-state fMRI analysis in Method 2). The dMRI sequence was a single-shot spin echo EPI sequence acquired in 30 non-collinear directions, using the following parameters: TR = 10,300 ms, TE = 85 ms, FOV = 256 × 256 mm, slice thickness = 2 mm, acquisition matrix = 128 × 128, and voxel size = 2 × 2 × 2 mm. Sixty-one axial slices were acquired without gap, giving full brain coverage. For each slice, one image without diffusion weighting (b = 0) and 60 images with diffusion gradients were acquired, consisting of 30 images with b = 600 s/mm^2^ and 30 with b = 1200 s/mm^2^.

#### 2.3.2. dMRI Data Preprocessing and Tractography

The diffusion-weighted images were preprocessed using FMRIB Software Library, version 5.0.9 (https://fsl.fmrib.ox.ac.uk/fsl/fslwiki) [17]. First, we used FSL’s eddy_openmp to correct for eddy current-induced distortions and subject movement [18]. The “replace outliers” option was used to identify outliers due to subject motion and replace the slices by using a non-parametric prediction based on Gaussian process regression (GPR) [19].

To acquire the streamline density map between the hippocampus and the whole brain for each subject, bedpost was first used to generate a Bayesian estimate of the probability distribution of different directions at each voxel [20]. The hippocampus (left and right hippocampus separately) as the seed mask was linearly transformed into the native space of each subject. Then, the fiber tracking was performed using FSL’s probatrackx2 [21,22], which generated streamlines connecting voxels from an originating seed ROI to voxels in a target ROI. The following settings were used for tractography: simple Euler streamlining, number of samples per voxel = 5000, number of steps per samples = 2000, step length = 0.5 mm, loop check, curvature threshold = 0.2, subsidiary fiber volume fraction threshold = 0.01, and seed sphere sampling = 0. The minimum streamline density map of the hippocampus to the whole brain were normalized to the standard space, threshold at 50% and binarized for each subject. Then, the tractographies were summed across subjects to produce the final tract pathways as a group-level probability map.

### 2.4. Summarizing Results from Neuroimaging Analyses

The results from Method 1, 2, and 3 were mapped onto a standard brain using Surf Ice (https://www.nitrc.org/projects/surfice) and a standard head using MRIcroGL (http://www.mccauslandcenter.sc.edu/mricrogl) with the international 10–20 electroencephalography (EEG) system in MNI space. The MNI coordinates of the 10-20 EEG system were extracted from a previous study [23]. In addition, we also used the international standard scalp acupuncture lines and acupoints to facilitate identifying the locations of the new scalp acupuncture protocol.

## 3. Results

### 3.1. Meta-Analysis Results

**Figure 1:**
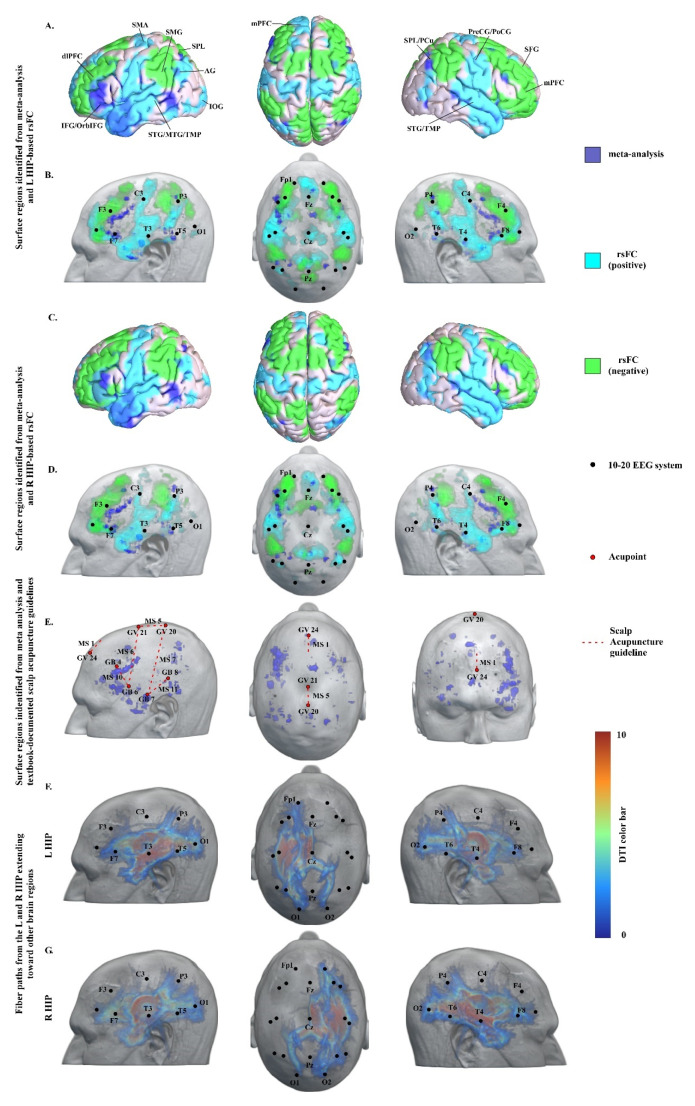
Potential targets for scalp stimulation for dementia identified from imaging study and acupuncture guidance. (**A**,**B**) Surface regions identified from meta-analysis and L HIP-based rsFC. (**C**,**D**) Surface regions identified from meta-analysis and R HIP-based rsFC. (**E**) Surface regions identified from meta-analysis and textbook-documented scalp acupuncture guidelines. (**F**,**G**) Fiber paths from the L and R HIP extending toward other brain regions. Abbreviations: L, left; R, right; HIP, hippocampus; rsFC, resting state functional connectivity; MTG, middle temporal gyrus; STG, superior temporal gyrus; IFG, inferior frontal gyrus; OrbIFG, orbital inferior frontal gyrus; dlPFC, dorsolateral prefrontal cortex; SMA, supplementary motor area; SPL, superior parietal lobule; PCu, precuneus; IOG, inferior occipital gyrus. TMP, temporal pole; SFG, superior frontal gyrus; mPFC, medial prefrontal cortex; PreCG, precentral gyrus; PoCG, postcentral gyrus; AG, angular gyrus; and SMG, supramarginal gyrus; MS, microsystem and scalppoints; MS 1, Ezhongxian; MS 5, Dingzhongxian; MS 6, Dingnieqianxiexian; MS 7; Dingniehouxiexian; MS 10, Nieqianxian; MS 11, Niehouxian; GV 20, Baihui; GV 21, Qianding; GV 24, Shenting; GB 4, Hanyan; GB 6, Xuanli; GB 7, Qubin; GB 8, Shuaigu.

Twelve regions on the brain surface were identified from the uniformity test map of the meta-analysis (Table 1). These brain regions were the left middle temporal gyrus (MTG)/inferior temporal gyrus (ITG), the bilateral superior temporal gyrus (STG)/superior temporal pole (STP)/middle temporal pole (MTP), the bilateral inferior frontal gyrus (IFG)/orbital inferior frontal gyrus (OrbIFG), the left inferior occipital gyrus (IOG), the right rostral prefrontal cortex (rPFC)/superior medial frontal gyrus (SupMFG), the left dorsolateral prefrontal cortex (dlPFC)/middle frontal gyrus (MFG)/triangular inferior frontal gyrus (TriIFG), the right supplementary motor area (SMA), and the right superior parietal lobule (SPL)/precuneus (PCu) (Table 1, Figure 1A,E). The 10-20 EEG system coordinates corresponding to the center of these regions were located approximately at F3, F4, F7, F8, T3, T4, T5, T6, Fz, and Pz (Figure 1B,D). Detailed whole brain results from the meta-analysis can be found in Table S2.

### 3.2. Resting-State Functional Connectivity Analysis Results

Seed-based resting-state functional connectivity analysis using the left and right hippocampi as seeds produced similar results. Specifically, we found that the brain surface regions, including the left superior frontal gyrus (SFG), SupMFG, dlPFC, the right PCu, SMA, precentral gyrus (PreCG) and postcentral gyrus (PoCG), as well as the bilateral STG, temporal pole (TMP), angular gyrus (AG) and IOG, were positively correlated with the left hippocampus. The bilateral mPFC, dlPFC, SFG and SPL, as well as the right operculum, were negatively correlated with the left hippocampus (Table 2, Figure 1A,B). Similarly, the left IOG, the right SOG, SupMFG, and mPFC, as well as the bilateral STG, MTG, ITG, TMP, SFG, PreCG, AG and MOG, were positively correlated with the right hippocampus. The left SPL, PoCG and PCu, the right mPFC, dlPFC, SMA, SFG and the cuneus, as well as the bilateral MFG, SMG, and AG, were negatively correlated with the right hippocampus (Table 3, Figure 1C,D). 10-20 EEG system coordinates corresponding to the center of these regions can be found in Figure 1B,D.

### 3.3. DTI Data Analysis Results

The goal of probabilistic tractography is to obtain an anatomic connectivity index along a white matter pathway that reflects fiber organization, which has been recognized as sensitive to pathological abnormalities [24]. Our results from probabilistic tractography revealed that the fiber paths from the left and right hippocampi extending toward brain surface regions were located approximately at F3, F4, P3, P4, O1, and O2 in the 10-20 EEG system (Figure 1F,G).

### 3.4. Neuroimaging-Based Scalp Acupuncture Protocol

**Figure 2:**
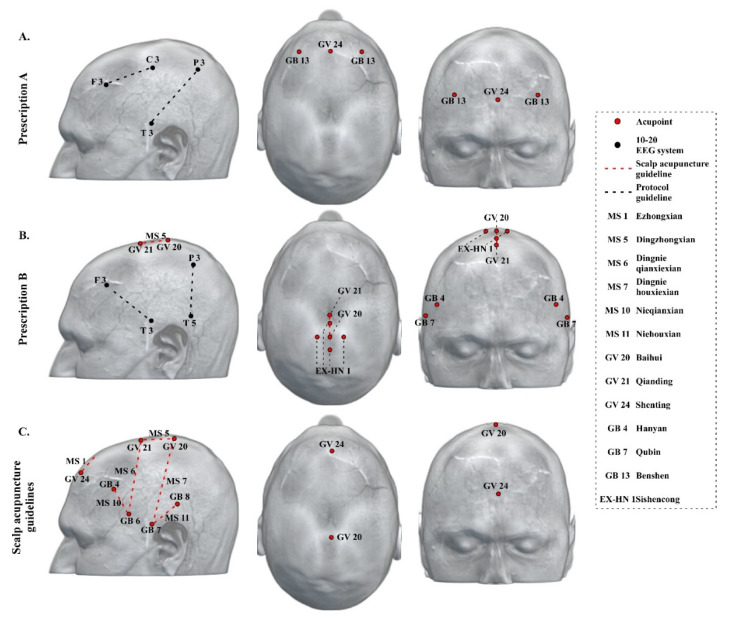
Neuroimaging-based scalp acupuncture prescription for dementia. (**A**) Prescription A, F3 to C3, T3 to P3, Shenting (GV 24), and bilateral Benshen (GB 13). B. Prescription (**B**) F3 to T3, P3 to T5, MS 5, Sishencong (EX-HN 1), bilateral Hanyan (GB 4), and Qubin (GB 7). (**C**) Textbook-documented scalp acupuncture guidelines. MS 1 (Ezhongxian, the middle line of forehead), 1 cun long from Shenting (GV 24) straight downward along the meridian; MS 5 (Dingzhongxian, the middle line of vertex), from Baihui (GV 20) to Qianding (GV 21) along the midline of head; MS 6 (Dingnieqianxiexian, the anterior oblique line of vertex-temporal), from Qianding (GV 21) obliquely to Xuanli (GB 6); MS 7 (Dingniehouxiexian, the posterior oblique line of vertex-temporal), from Baihui (GV 20) obliquely to Qubin (GB 7); MS 10 (Nieqianxian, anterior temporal line), from Hanyan (GB 4) to Xuanli (GB 6); and MS 11 (Niehouxian, posterior temporal line), from Shuaigu (GB 8) to Qubin (GB 7). Abbreviation: MS, microsystem and scalppoints; GV, Governor Vessel; GB, Gallbladder meridian.

Based on the findings from Methods 1–3, we proposed a neuroimaging-based scalp acupuncture prescription for dementia. In prescription A, we prescribed treatment lines from F3 to C3, and from T3 to P3, as well as acupoints Shenting (GV 24), and bilateral Benshen (GB 13). In prescription B, we prescribed treatment lines from F3 to T3, from P3 to T5, and the MS 5 (Dingzhongxian, the middle line of vertex, from Baihui (GV 20) to Qianding (GV 21) along the midline of head), as well as acupoints Sishencong (EX-HN 1), and bilateral Xuanli (GB 4) and Qubin (GB 7) (Figure 2A,B). The two prescriptions should be applied alternately to avoid treatment resistance.

## 4. Discussion

Scalp acupuncture is an acupuncture modality based on brain anatomy and function. Recently, the application of brain imaging tools has led to remarkable progress in the research of various neurological and psychotropic diseases, thereby building a foundation for development of scalp acupuncture protocols. In this study, we explored a new method for developing/updating the protocols for scalp acupuncture. Specifically, we integrated meta-analysis, resting-state functional connectivity, and DTI to identify potential locations for scalp acupuncture for treatment of dementia. We found brain regions such as the prefrontal cortex (mPFC/dlPFC), the MTG, the STG, the TMP, the SMA, the IOG, and the PCu to be the target areas of scalp acupuncture for treatment of dementia. Furthermore, we also used the 10-20 EEG system, the International standard scalp acupuncture system, and scalp acupoints to facilitate the location of these target areas on the scalp.

### 4.1. Key Regions/Locations in the Neuroimaging-Based Scalp Acupuncture Prescription

In this study, we identified multiple surface areas to be the target regions for scalp acupuncture. Our findings are consistent with the physiology/function of these brain regions.

For instance, the prefrontal cortex has been proven to play a crucial role in the pathophysiology of dementia. Burgmans and colleagues found that prefrontal atrophy was highly associated with dementia and can be considered an important predictor of the disease [25]. More specifically, the dlPFC is highlighted in brain stimulation treatments for dementia. Previous studies have demonstrated the dlPFC as a promising target commonly used in transcranial magnetic stimulation (TMS) to improve the cognitive performance of dementia patients [26,27,28]. Another study applied anodal transcranial direct current stimulation (tDCS) on the dlPFC in patients with mild vascular dementia and observed clinical benefits in patients’ short-term memory, verbal working memory, as well as executive control [29]. Further imaging studies suggest that stimulating the dlPFC using a neuromodulation method may work through changes in dopamine concentration [30].

Both the PCu and the mPFC are core regions of the default mode network (DMN) [31,32]. Recently investigators found that the DMN is particularly relevant for dementia, because the DMN regions are vulnerable to neurodegenerative pathological changes, including atrophy, deposition of the amyloid protein, and reduced glucose metabolism [33,34]. In addition, Wang and colleagues have reported that the hippocampus, which plays a key role in the pathology of dementia, showed decreased functional connectivity with the mPFC and the PCu in patients with Alzheimer’s disease [35]. We also found that the PCu showed significant functional connectivity with the hippocampus. Taken together, these findings provide direct support for using the PCu and the mPFC as targets for dementia [36,37,38].

Studies have indicated that individuals with dementia tend to have communication and memory difficulties. Studies have found that dementia, especially semantic dementia, is often accompanied by abnormalities of the temporal lobe [39,40]. The relatively clear diagnosis and pathological homogeneity of semantic dementia make the temporal regions promising targets for innovative interventions [41]. Our results from both meta-analysis and the hippocampus functional connectivity analysis endorsed the role of the MTG, STG, and TMP. Thus, these regions are included in the scalp acupuncture prescription for treating dementia, and particularly for treatment of semantic dementia, or individuals with communication difficulties.

The SMA is another crucial region associated with memory that is typically spared in the early stage of dementia. Neuroimaging studies on both healthy and brain-damaged individuals have shown that the SMA is part of a widespread frontoparietal network underlying working memory [42]. A recent study found that damage of the SMA is associated with working memory impairment [43].

Based on above neuroimaging findings, we have proposed a new prescription including five treatment lines: from F3 to C3, from T3 to P3, from F3 to T3, from P3 to T5, and MS 5. In addition, the location of Shenting (GV 24), Sishencong (extra points on the head and neck (EX-HN) 1), and the bilateral Hanyan (GB 4), Qubin (GB 7) and Benshen (GB 13) also overlapped the neuroimaging results. Thus, these scalp acupoints were incorporated in our prescription.

### 4.2. Current Scalp Acupuncture Protocols for Dementia and Differences Compared to the Neuroimaging-Based Scalp Acupuncture Protocol

At present, there is no international standard guidance for scalp acupuncture therapy for dementia. We searched acupuncture textbooks used by Traditional Chinese Medicine Universities to summarize the scalp acupuncture treatment guidelines and commonly used scalp acupoints for dementia.

According to the scalp acupuncture treatment guidance from the textbook “Acupuncture Therapeutics” (Second edition, China Press of Traditional Chinese Medicine, 2007), the middle line of forehead (Ezhongxian, 1 cun long from Shenting (Governor Vessel (GV) 24) straight downward along the meridian, MS 1), the middle line of vertex (Dingzhongxian from Baihui (GV 20) to Qianding (GV 21) along the midline of head, MS 5), anterior temporal line (Nieqianxian, from Hanyan (Gallbladder meridian (GB) 4) to Xuanli (GB 6), MS 10), and posterior temporal line (Niehouxian, from Shuaigu (GB 8) to Qubin (GB 7). MS 11) were recommended for treatment of dementia.

Based on the textbook “Clinical Acupuncture” (First edition, China Science Publishing & Media Ltd. (CSPM), 2015), MS 1, MS 5, the anterior oblique line of vertex-temporal (Dingnieqianxiexian, from Qianding (GV 21) obliquely to Xuanli (GB 6), MS 6), and the posterior oblique line of vertex-temporal (Dingniehouxiexian, from Baihui (GV 20) obliquely to Qubin (GB 7), MS 7) were selected for scalp acupuncture guidelines in treating dementia. Please see Figure 2C for a summary of current scalp acupuncture prescription for dementia.

Differences and similarities exist in the comparison between the neuroimaging-based prescription and the textbook-documented guidelines. Specifically, both protocols used the mPFC, SMA, IFG, PreCG, PoCG, STG, MTG, and TMP regions as stimulation targets. However, the neuroimaging-based prescription incorporates several additional areas, including the dlPFC, MFG, SFG, PCu, SPL, and IOG. We believe that these additional brain regions reflect our enhanced understanding on the brain network involved in dementia and, thus, should be incorporated into the current scalp acupuncture protocols.

### 4.3. Additional Application of the Neuroimaging-Based Scalp Acupuncture Prescription

It is worth noting that the locations identified in this study may not be limited to acupuncture and can also be applied to neuromodulation methods, such as transcranial magnetic stimulation (TMS), transcranial direct current stimulation (tDCS), and transcranial alternating current stimulation (tACS).

In addition, the scalp acupuncture prescription we proposed may also be integrated with traditional acupuncture theory (Traditional Chinese Medicine-based acupuncture). A crucial characteristic of traditional acupuncture treatment is that it is consistent with syndrome differentiation. Thus, the acupoints that are selected for traditional acupuncture should also consider individual diversities of patients. The protocol needs to be adjusted according to the individual’s symptoms and status at the time of use.

### 4.4. Limitation and Future Directions

There are several limitations to our study. First, we combined different methods to identify potential surface targets for dementia. Regions identified from Method 1 are believed to be associated with dementia pathophysiology directly, and regions identified from Method 2 and 3 are surface cortex with strong functional or anatomical connection with the key deep structure brain region associated with dementia. Understanding the derivative of these locations may help researchers choose target regions during clinical practice. Second, the aim of this study was to explore the potential brain surface targets for dementia; the application and optimization of different treatment techniques to target/modulate these brain regions is beyond the scope of this manuscript. Further, we only used the hippocampus as the region of interest in functional connectivity and DTI analyses due to its crucial role in memory and dementia. Other deep brain structures may also play an important role in dementia and may be used as the region of interest to conduct functional and anatomical connectivity analysis to extend the neuroimaging-based acupuncture protocol. Thirdly, dementia is an umbrella term used to describe clinical syndromes of progressive cognitive decline, which included subtypes such as Alzheimer’s disease, Lewy body dementia, frontotemporal dementia, vascular dementia, etc. Each of these disorders may be associated with different pathology. This study focused on dementia as a set of symptoms, including memory loss and difficulties with thinking, problem solving or language, which commonly appear in subtypes of dementia, and thus, may have common potential functional/structural mechanisms. Future studies are needed to compare the potential targets for different disorders. In addition, studies show that dementia may somehow alter the connectivity, but the extent may differ based on specific disorders and development stages (early vs. late stages). We decided to use healthy subjects, which may provide a more unbiased evaluation in Method 2 and 3. Finally and also most importantly, our findings need to be validated with clinical trials/studies on patients with dementia.

## 5. Conclusions

In conclusion, we have developed a new scalp acupuncture protocol for dementia based on brain imaging data analyses. Our study may shed light on the development of scalp acupuncture, particularly in the domain of identifying stimulation locations.

## Figures and Tables

**Table 1:** Coordinates of dementia-associated surface regions identified from meta-analysis.

Cluster ID	Cluster Size	Peak T	Peak Coordinates			Brain Regions
			x	y	z	
1	71	6.32	−46	4	−42	L MTG/ITG
2	30	4.79	−46	10	−26	L STG/MTG/STP/MTP
3	107	7.85	−56	−10	−18	L MTG
4	47	7.09	46	10	−24	R STG/MTG/STP
5	106	6.32	−38	22	−4	L IFG/OrbIFG
6	37	5.56	−44	−58	−12	L IOG
7	33	5.56	34	24	−10	R IFG/OrbIFG
8	44	5.56	−50	34	−4	L IFG/OrbIFG
9	49	6.32	4	50	0	R rPFC/SupMFG
10	292	7.09	−46	32	16	L dlPFC/MFG/IFG/TriIFG
11	57	6.32	10	12	46	R SMA
12	35	6.32	26	−64	50	R SPL/PCu

Abbreviations: L, left; R, right; MTG, middle temporal gyrus; ITG, inferior temporal gyrus; STG, superior temporal gyrus; STP, superior temporal pole; MTP, middle temporal pole; MFG, middle frontal gyrus; IFG, inferior frontal gyrus; OrbIFG, orbital inferior frontal gyrus; TriIFG, triangular inferior frontal gyrus; SupMFG, superior medial frontal gyrus; rPFC, rostral prefrontal cortex; dlPFC, dorsolateral prefrontal cortex; SMA, supplementary motor area; SPL, superior parietal lobule; PCu, precuneus; IOG, inferior occipital gyrus.

**Table 2:** Dementia-associated surface regions identified from L hippocampus-based resting-state functional connectivity (rsFC).

rsFC	Brain Regions	Cluster	Peak	MNI Coordinates		
		Size	T	x	y	z
Positive	L STG/MTG/TMP	8175	15.56	−62	−2	−2
	R PCu	2098	9.58	6	−54	28
	L SupMFG/dlPFC	2744	14.66	−2	48	−14
	R STG/TMP/operculum	6228	11.86	56	−2	−12
	R PreCG/SMA/PoCG	1483	8.39	2	−16	54
	L SFG	145	6.23	−18	32	48
	L AG	282	5.34	−46	−70	30
	R AG	132	5.26	48	−60	26
	L IOG	160	5.24	−22	−88	−4
	R IOG	43	4.63	50	−76	4
Negative	R SPL/AG	1922	14.08	46	−42	40
	R mPFC/dlPFC	4098	12.70	38	34	38
	L SPL/SMG	1669	9.00	−36	−50	48
	L mPFC/dlPFC	2542	7.99	−36	30	30
	L SPL/cuneus	1879	7.40	−8	−66	50
	R SFG	65	5.78	6	34	38
	L SFG	283	5.72	−24	8	68
	R operculum	99	5.65	50	12	10

Notes: Results are significant at cluster P_FDR_ < 0.05 corrected at whole-brain level. Abbreviations: L, left; R, right; rsFC, resting-state functional connectivity; MTG, middle temporal gyrus; STG, superior temporal gyrus; TMP, temporal pole; MFG, medial frontal gyrus; SFG, superior frontal gyrus; SupMFG, superior medial frontal gyrus; mPFC, medial prefrontal cortex; dlPFC, dorsolateral prefrontal cortex; PreCG, precentral gyrus; PoCG, postcentral gyrus; SMA, supplementary motor area; SPL, superior parietal lobule; PCu, precuneus; AG, angular gyrus; IOG, inferior occipital gyrus; SMG, supramarginal gyrus.

**Table 3:** Dementia-associated surface regions identified from R hippocampus -based resting-state functional connectivity (rsFC).

rsFC	Brain Regions	Cluster	Peak	MNI Coordinates		
		Size	T	x	y	z
Positive	R MTG/STG/TMP	5785	12.22	56	−4	−26
	R PCu	2182	11.90	14	−36	4
	L STG/TMP/MTG	4615	10.63	−60	−2	−4
	R SFG/SupMFG/mPFC	1503	9.88	4	34	−10
	L ITG	177	7.39	−34	−36	−14
	R ITG/AG/MTG/MOG/IOG	1100	7.03	50	−46	−28
	L PreCG	60	6.21	−36	−20	72
	R SFG	98	6.05	20	36	48
	L AG/MOG	133	5.57	−40	−66	22
	L ITG	53	4.99	−48	−58	−14
	R PreCG	85	4.76	38	−12	68
	R SFG	56	4.67	4	52	20
	L SFG	61	4.52	−12	58	10
	L SOG	49	4.40	−20	−90	12
	L PreCG	70	4.39	−6	−26	62
	R PreCG	48	4.30	12	−18	78
Negative	R MFG/mPFC/dlPFC	2525	14.03	34	54	22
	L SMG/SPL/AG	1759	12.14	−48	−46	40
	L MFG	3690	9.75	−30	48	−2
	R SMG/AG	1374	9.08	50	−42	38
	R SFG/SMA/MFG	1263	8.60	16	20	60
	L PCu	190	6.11	−2	−52	70
	R cuneus	135	4.73	8	−80	28
	L SPL/PoCG	42	4.33	−20	−56	72

Notes: Results are significant at cluster P_FDR_ < 0.05 corrected at whole-brain level. Abbreviations: L, left; R, right; rsFC, resting-state functional connectivity; MTG, middle temporal gyrus; STG, superior temporal gyrus; TMP, temporal pole; MFG, medial frontal gyrus; SFG, superior frontal gyrus; SupMFG, superior medial frontal gyrus; mPFC, medial prefrontal cortex; dlPFC, dorsolateral prefrontal cortex; PreCG, precentral gyrus; PoCG, postcentral gyrus; SMA, supplementary motor area; ITG, inferior temporal gyrus; MTG, middle temporal gyrus; SPL, superior parietal lobule; PCu, precuneus; AG, angular gyrus; IOG, inferior occipital gyrus; MOG, middle occipital gyrus; SOG, superior occipital gyrus; SMG, supramarginal gyrus.

## Supplementary Materials

The following are available online at https://www.mdpi.com/2077-0383/9/8/2477/s1, Table S1: List of 142 studies extracted from Neurosynth under search term “dementia” on April 16, 2020; Table S2: Coordinates of dementia-associated regions identified from meta-analysis.
